# THE CONFLUENCE OF SOCIAL ISOLATION, LONELINESS, AND ITS ASSOCIATION WITH COGNITIVE DECLINE

**DOI:** 10.1093/geroni/igae098.2546

**Published:** 2024-12-31

**Authors:** Joseph Svec, Jinshil Hyun, JeongEun Lee

**Affiliations:** St. Joseph’s University, Patchogue, New York, United States; Albert Einstein College of Medicine, Bronx, New York, United States; Iowa State University, Ames, Iowa, United States

## Abstract

Numerous studies indicate that social isolation and loneliness are crucial predictors of cognitive decline among older adults. Specifically, cognitive functioning (i.e. memory) is positively linked with robust social connections (Kang and Oremus, 2023). While social isolation and loneliness have gained considerable attention in recent literature, there remains substantial ambiguity in the distinct impacts of isolation and loneliness where both are often treated as part of a similar construct or examined separately (Boss et al., 2015). Given the potential ramifications for cognitive functioning, as well as implications for pragmatic solutions regarding social, environmental, and technological approaches, this study seeks to examine and investigate both the independent and concurrent effects of isolation and loneliness on cognitive functioning. Using data from eight waves of Health and Retirement Study (HRS) data, we utilize fixed effects linear regression models of older adults’ (60 years or older) immediate and delayed memory recall score on measures of social isolation, feelings of loneliness, as well as their interactions (N = 44,170). Analyses emphasize within-person associations of memory and covariates. We observe significant and negative associations of isolation (coeff = -0.02, p < 0.01) and loneliness (coeff = -0.08, p < 0.01) for both immediate and delayed recall score. However, a positive and significant interaction of loneliness and isolation indicates that feelings of loneliness is more strongly associated with immediate and delayed memory when social isolation is low (frequently meets with family or friends) – when social isolation is high, the association of loneliness and cognitive functioning is limited.

